# Supplementary figures and images for: Reassessing the adrenomedullin scavenging function of ACKR3 in lymphatic endothelial cells
Source: PLoS One. 2023 May 30;18(5):e0285597. doi: 10.1371/journal.pone.0285597 (PMC10228790; doi:10.1371/journal.pone.0285597)

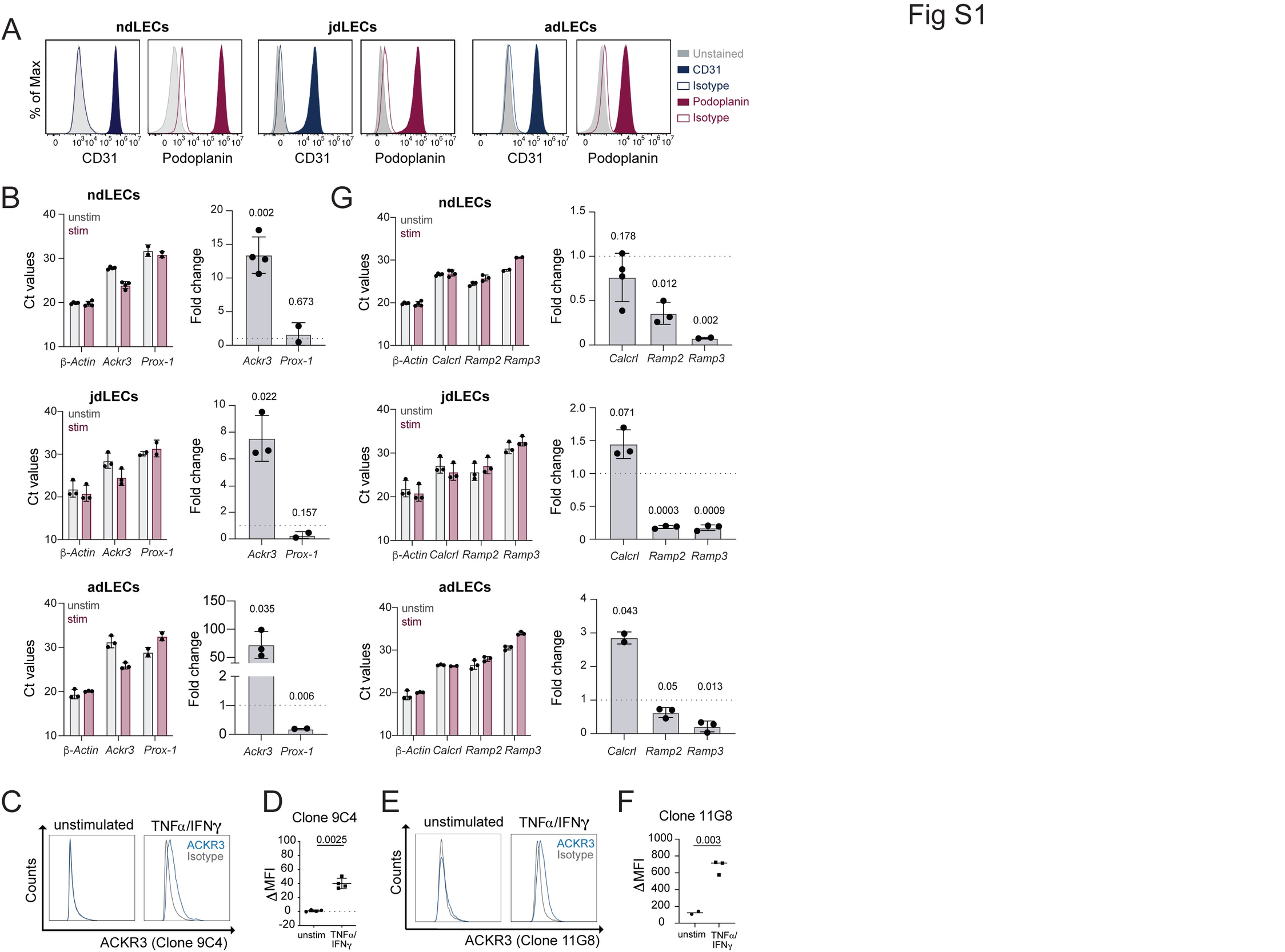

Supplement: S1 Fig — (A) FACS analysis showing expression of CD31 and podoplanin in ndLECs, jdLECs and adLECs. (B) qRT-PCR was performed to investigate Ackr3 mRNA expression in steady-state (unstim) and TNFα/ IFN® stimulated (stim) ndLECs, jdLECs and adLECs. Prox-1 levels were determined for comparison. CT values are shown on the left and resulting fold-changes on the right. (C-F) ACKR3 protein expression was not detectable in resting ndLECs (unstim) by flow cytometry, but could be detected on the ndLEC cell surface after TNFα/ IFNγstimulation (stim). Staining was performed with two different anti-ACKR3 antibodies, i.e. with (C, D) clone 9C4 and (E, F) clone 11G8. Representative histograms are shown in (C,E) and quantifications of 3–4 independent experiments in (D,E). Each dot represents the ΔMFI value (normalized to the isotype control) from one staining. Student’s t-test. (G) qRT-PCR was performed to investigate Calcrl, Ramp2 and Ramp3 mRNA expression in steady-state (Ctrl) and TNFα/ IFN® stimulated (stim) ndLECs, jdLECs and adLECs. Data from 1 RNA extraction and 3 technical replicates are shown in (B,G). Statistical analysis performed in (B,G): One sample t test. (TIF) [file pone.0285597.s001.tif]

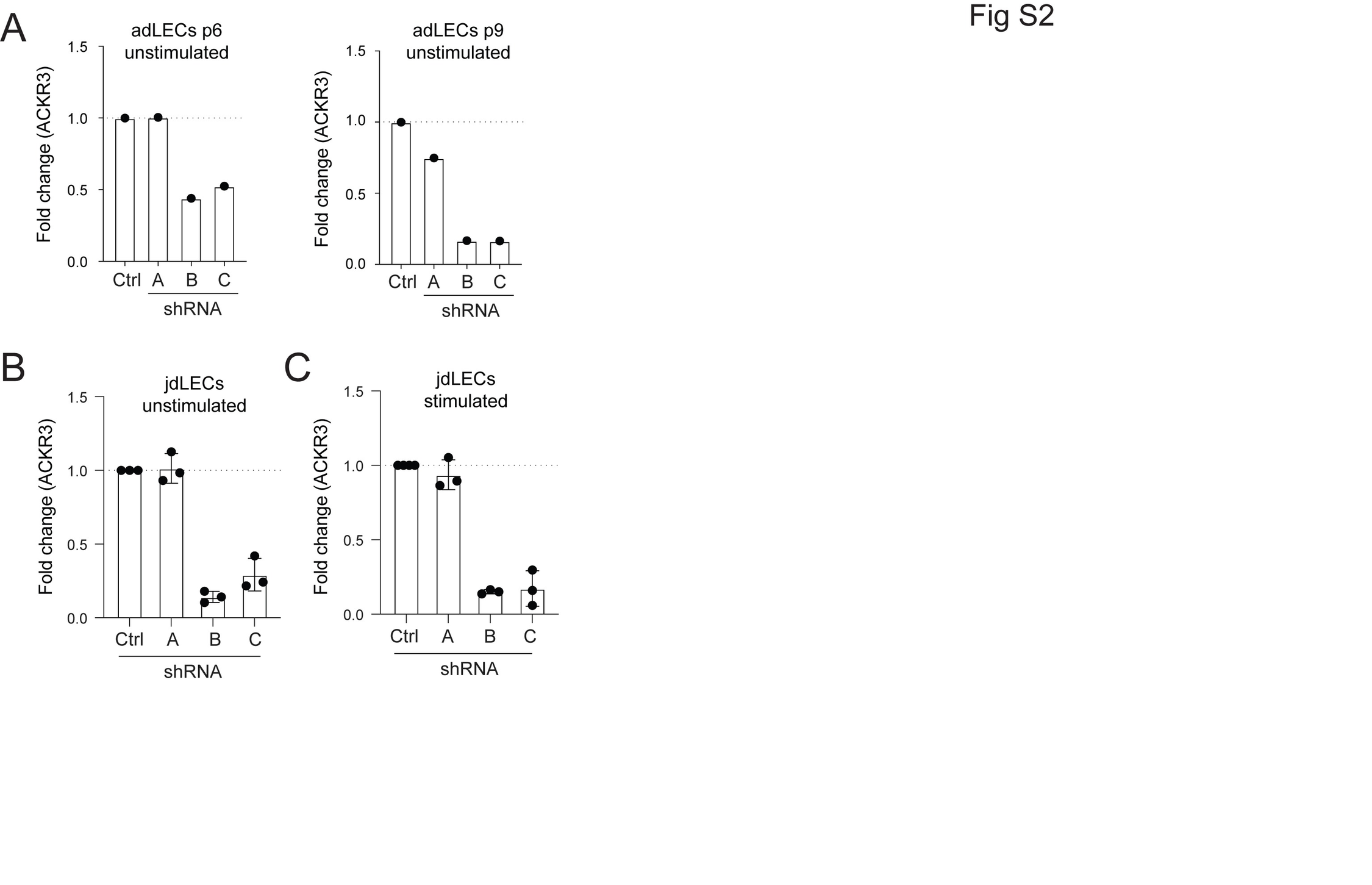

Supplement: S2 Fig — Validation of shRNA-mediated ACKR3 knockdown in lentivirally transduced and subsequently sorted adLECs and jdLECs, in comparison to untransduced (Ctrl) or scrambled RNA-transduced (shRNA Ctrl) cells. (A) Knockdown efficiency in steady-state adLECs was analysed at p6, shortly, after sorting and p9, after all experiments were performed. (B) Knockdown efficiency in resting jdLECs at p8. (C) Knockdown efficiency in TNFα/IFN® stimulated shRNA-transduced jdLECs at p8. Each data point represents the normalized relative expression calculated from three technical replicates per repetition. (TIF) [file pone.0285597.s002.tif]

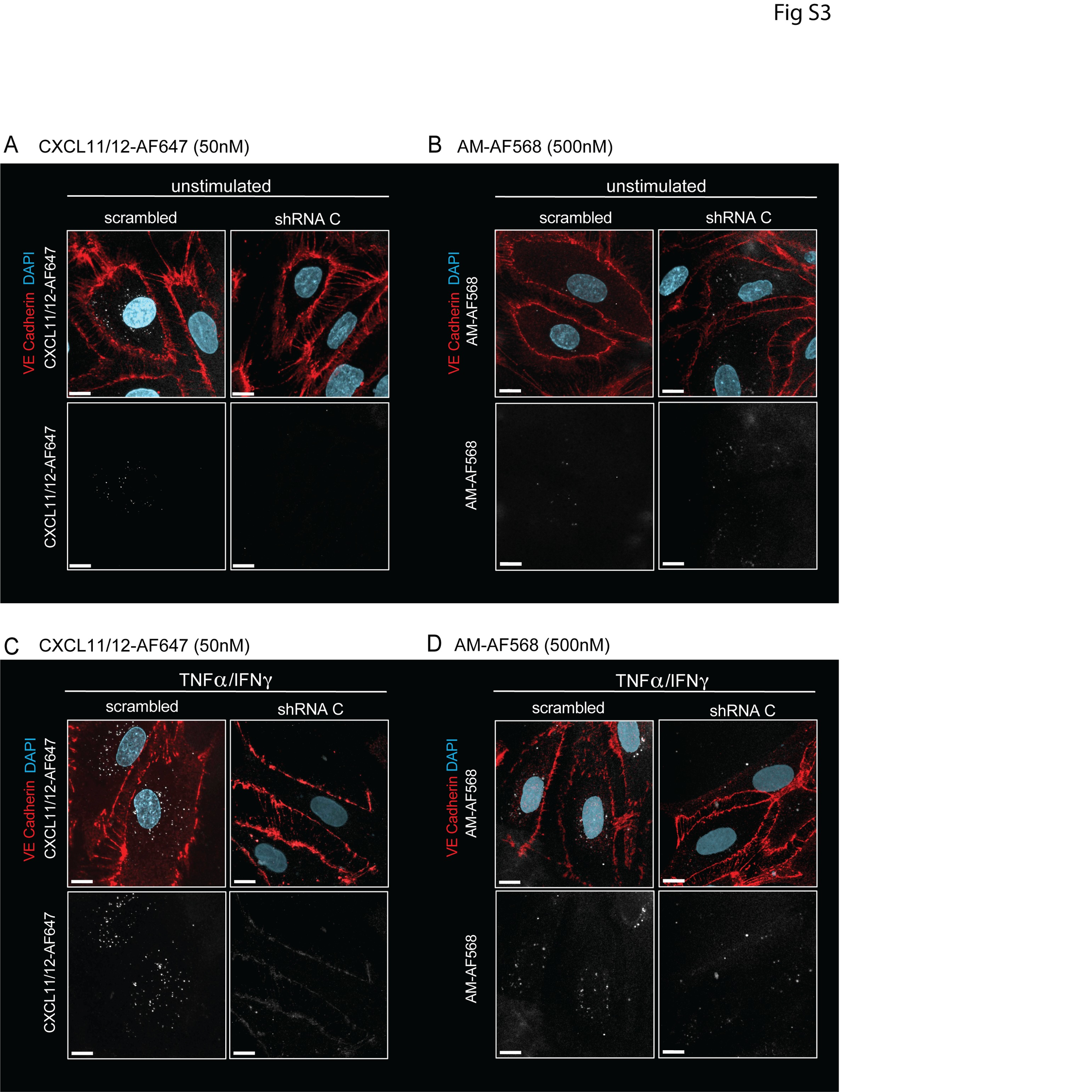

Supplement: S3 Fig — Sorted jdLECs (p7), transduced with either scrambled control shRNA or ACKR3-targeting scRNA construct C, were first starved, then stimulated for 24h with 20ng TNFα/ IFN® or left untreated. Uptake assays were performed with AM-AF568 (500nM) or CXCL11/12-AF647 (50 nM). (A, B) Representative images of (A) CXCL11/12-AF647 and (B) AM-AF568 uptake performed in unstimulated (Ctrl) jdLECs. (C, D) Representative images of 2 uptake experiments performed with (C) CXCL11/12-AF647 and (D) AM-AF568 in TNFα/IFNγ stimulated jdLECs. Scale bars: 10μm. (TIF) [file pone.0285597.s003.tif]

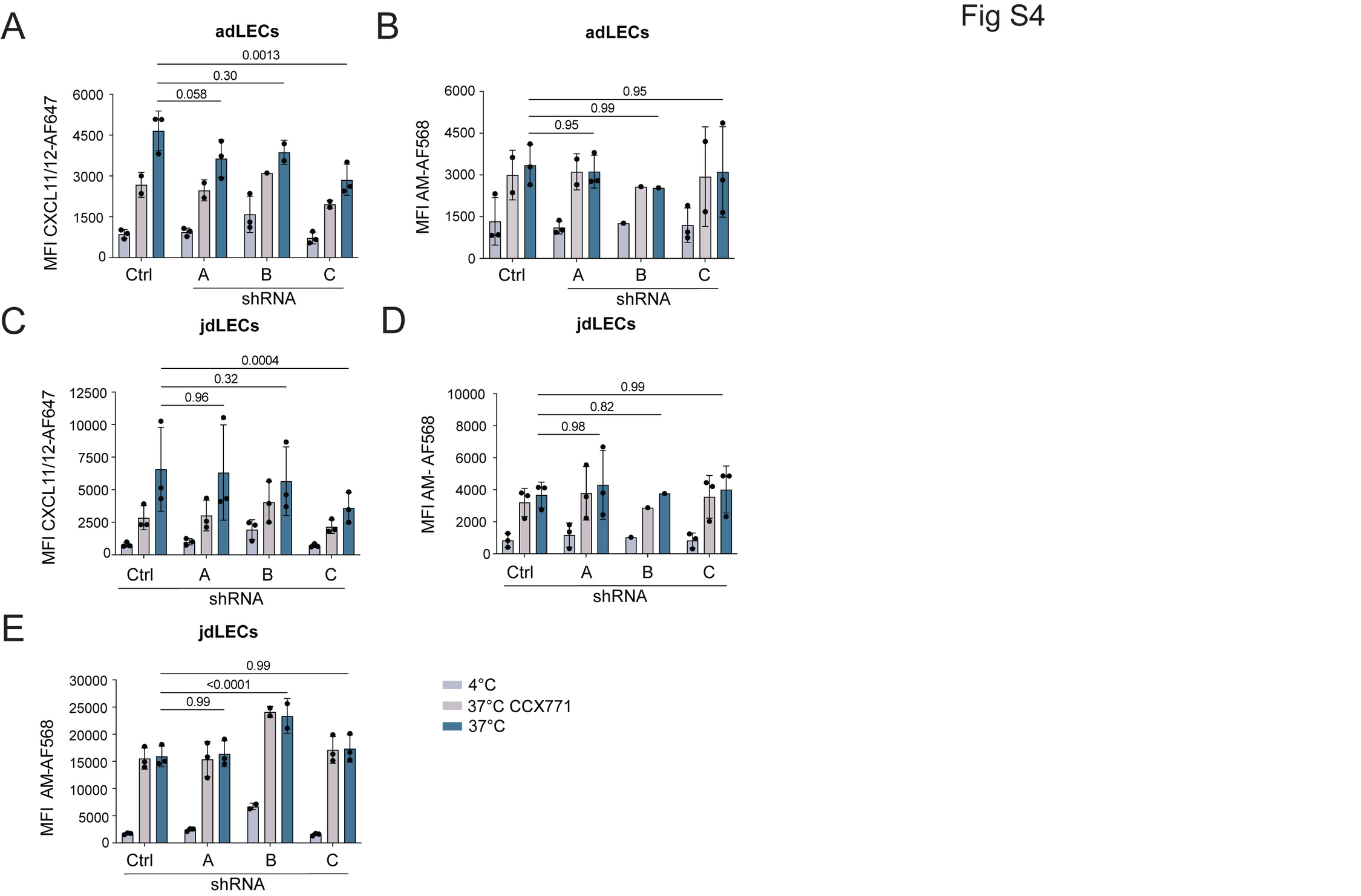

Supplement: S4 Fig — adLECs and jdLECs were transduced with ACKR3-specific shRNA constructs (A: no knockdown, B,C; 50–80% knockdown–see S2 Fig), scrambled shRNA (shRNA Ctrl) or untransduced control LECs (Ctrl). For uptake assays, cells were incubated with either CXCL11/12-AF647 (50nM) or AM-AF568 (50nM) and uptake analysed by FACS. While CXCL11/12-AF647 scavenging correlated with ACKR3 knockdown in (A) adLECs and (C) jdLECs, no impact of ACKR3 levels on AM-AF568 scavenging was observed in (B) adLECs and (D) jdLECs. (E) Also, when performing the experiment with 500nM AM-AF568, no impact of ACKR3-knockdown on scavenging was observed. Data from three or four independent experiments are shown as mean ±SD. Each data point represents one replicate. Statistics: Two-way ANOVA, followed by Tukey’s multiple comparison test (C). A mixed effects model, followed by Tukey’s multiple comparison test, was applied in all other cases, due to incomplete repeated measures-pairing (A, B, D, E). A p-value of p≥0.05 was considered not significant (ns). (TIF) [file pone.0285597.s004.tif]

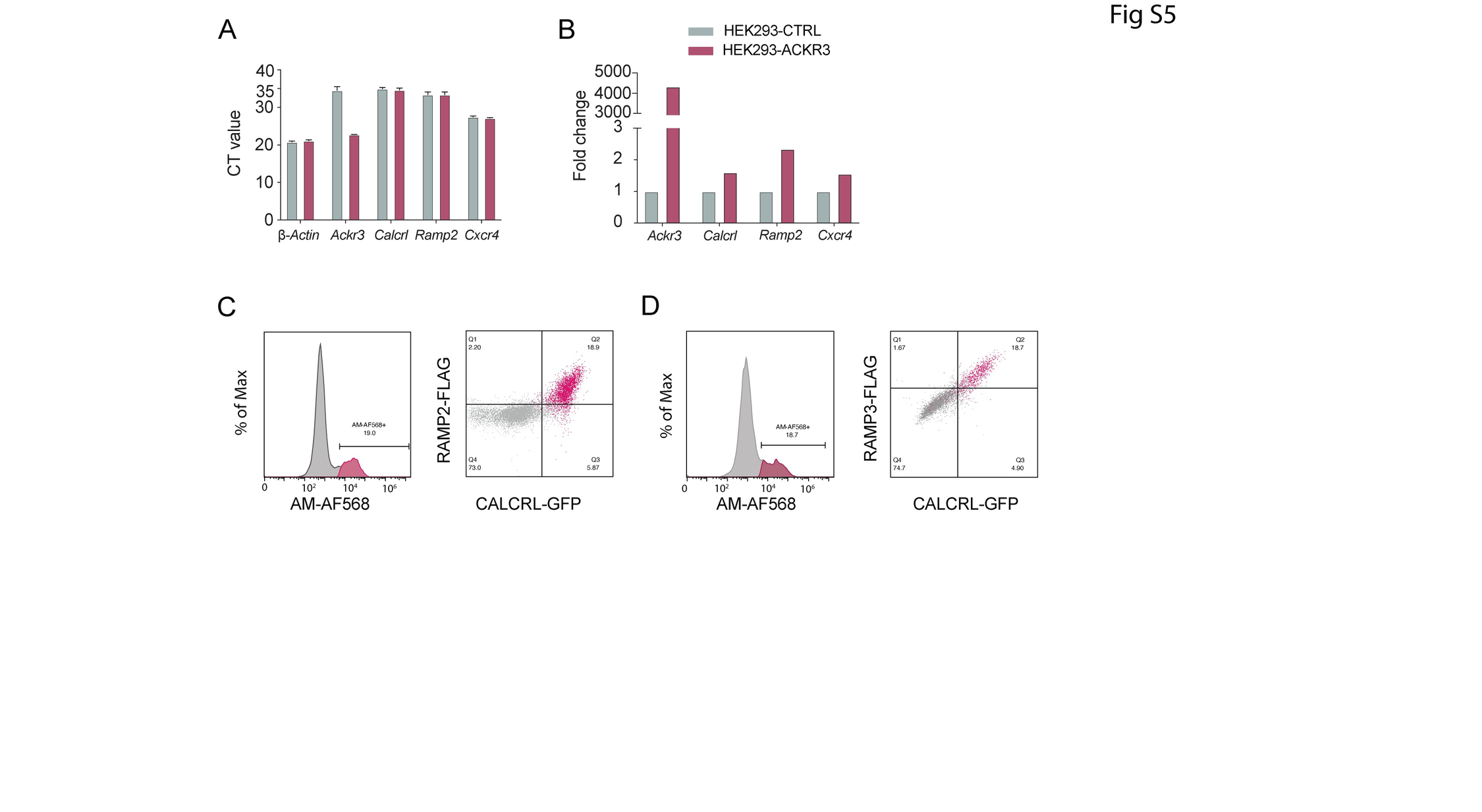

Supplement: S5 Fig — (A, B) Analysis of mRNA expression in untransfected HEK293 control (HEK293-CTRL) and HEK293-ACKR3 cells indicated no endogenous expression of Ackr3, Calcrl or Ramp2 in HEK293-CTRL cells. (A) Comparative mRNA expression between HEK293-CTRL and HEK293-ACKR3 cells. Data are shown as mean ±SD. (B) Fold change. Values from one qRT-PCR experiment, with three technical replicates are shown. (C, D) FACS plots of uptake experiments performed in presence of 50nM AM-AF568 in HEK293-CTRL and HEK293-ACKR3 cells transiently transfected with (C) CALCLRL-GFP and RAMP2-FLAG or with (D) CALCLRL-GFP and RAMP3-FLAG. Back-gating (dot plot on right) demonstrated that AM-AF568 was exclusively scavenged by cells co-expressing both constructs. Representative Dot plots of one out of four independent experiments are shown in (C, D). (TIF) [file pone.0285597.s005.tif]
