# Supplementary material for: Reassessing the adrenomedullin scavenging function of ACKR3 in lymphatic endothelial cells
Source: PLoS One. 2023 May 30;18(5):e0285597. doi: 10.1371/journal.pone.0285597 (PMC10228790; doi:10.1371/journal.pone.0285597)
Supplement: Attachment — Submitted filename: Reviewer 1.pdf [file pone.0285597.s006.pdf]

Cancel | Save and Close

PONE-D-23-00791
"Reassessing the adrenomedullin scavenging function of ACKR3 in lymphatic endothelial cells"
Original Submission

[redacted] **(Reviewer 1)**

**Reviewer Recommendation Term:**	Minor Revision
**Rate Review:**	0
**Custom Review Question(s):**	**Response**
**Comments to the Author**<br><br>1. Is the manuscript technically sound, and do the data support the conclusions?<br><br>The manuscript must describe a technically sound piece of scientific research with data that supports the conclusions. Experiments must have been conducted rigorously, with appropriate controls, replication, and sample sizes. The conclusions must be drawn appropriately based on the data presented.	Partly
2. Has the statistical analysis been performed appropriately and rigorously?	I Don't Know
3. Have the authors made all data underlying the findings in their manuscript fully available?<br><br>The PLOS Data policy requires authors to make all data underlying the findings described in their manuscript fully available without restriction, with rare exception (please refer to the Data Availability Statement in the manuscript PDF file). The data should be provided as part of the manuscript or its supporting information, or deposited to a public repository. For example, in addition to summary statistics, the data points behind means, medians and variance measures should be available. If there are restrictions on publicly sharing data—e.g. participant privacy or use of data from a third party—those must be specified.	Yes
4. Is the manuscript presented in an intelligible fashion and written in standard English?<br><br>PLOS ONE does not copyedit accepted manuscripts, so the language in submitted articles must be clear, correct, and unambiguous. Any typographical or grammatical errors should be corrected at revision, so please note any specific errors here.	Yes
5. Review Comments to the Author<br><br>Please use the space provided to explain your answers to the questions above. You may also include additional comments for the author, including concerns about dual publication, research ethics, or publication ethics. (Please upload your review as an attachment if it exceeds 20,000 characters)	This manuscript from the Halin laboratory has reassessed the established notion (Klein et al. Dev Cell 2014) that ACKR3 acts as a scavenger of the opioid peptide adrenomedullin (AM) in LECs. They have convincingly demonstrated that, in their hands, ACKR3 does not have scavenging activity on AM at physiologically relevant concentrations either in primary LECs (from multiple sources) or transfected cell lines. They have used a combination of approaches to demonstrate this, including knock down of ACKR3 and an ACKR3-specific inhibitor. The experiments are generally well controlled and unequivocal. The manuscript is well-written, appropriately discussed and the data generally are well presented. There are a few concerns regarding statistical analysis, rigour and data interpretation that should be addressed prior to publication however.<br><br>1) Figure 1A: data is from a single experiment. This should be done more than once at a minimum.<br><br>2) Figure 1A and 1B: quantification and statistical analysis should be provided here.<br><br>3) Figure 2C: a direct statistical comparison between the MFI of the '37C AM-AF568' condition between the unstim and TNFa/IFNg groups should be provided to support the claim made on line 354 of the results.<br><br>4) Figure 4E and 4F: To show that the signal measured for AM/chemokine uptake in these assays is specific, a negative control should be provided (e.g. cells at 4C)<br><br>5) Figure 4H and 4I: the 50nM group depicted in these plots appears to be identical to the data shown in Figure 2C. This group should be removed from 4H and 4I to avoid this duplication.<br><br>6) It is notable that the data shown in Figure 4D and 4E appear to suggest that the KD of ACKR3 using shRNA 'C' has actually inhibited AM-induced LEC proliferation. While this is clearly the opposite of what would be expected based on the data published by Klein et al. Dev Cell 2014, the authors should discuss what might underly this.<br><br>7) Some of the p values being reported in the manuscript do not seem to match the distribution of data points as presented. Figure 4F (Ctrl: Ctrl v AM p=0.0010); Supp Figure 4C (Ctrl 37C v shRNA C 37C p=0.0004). Please clarify how these values have been computed.<br><br>8) Statistical tests and p values should be provided in Supp Fig 1B and SF1G to support claims made in the manuscript.<br><br>Minor: human gene names should be italicised all caps not lower case for annotation of genes in qPCR
6. PLOS authors have the option to publish the peer review history of their article (what does this mean?). If published, this will include your full peer review and any attached files.<br><br>If you choose "no", your identity will remain anonymous but your review may still be made public.<br><br>**Do you want your identity to be public for this peer review?** For information about this choice, including consent withdrawal, please see our Privacy Policy.	No
**Confidential to Editor**<br><br>1. Do you have any potential or perceived competing interests that may influence your review? Please review our Competing Interests policy and declare any potential interests that you feel the Editor should be aware of when considering your review. If you have no competing interests, please write "I have no competing interests."	This is a solid study that requires only minor revisions
2. Did you receive any assistance in preparing this review (e.g. from a post-doc or graduate student)? If yes, please include their name below.	no
3. If accepted, do you think this submission should be highlighted on the PLOS ONE website? PLOS ONE does not evaluate manuscripts based on perceived significance or readership. We aim to provide tools for readers to filter and evaluate our publications. (optional)	No
**Do you want to get recognition for this review on a Web of Science researcher profile?**<br><br>If you opt in, your Web of Science profile will automatically be updated to show a verified record of this review in full compliance with the journal's review policy. If you don't have a Web of Science profile, you will be prompted to create a free account.	No

More Reviewer Details

Cancel | Save and Close
