# Supplementary material for: Reassessing the adrenomedullin scavenging function of ACKR3 in lymphatic endothelial cells
Source: PLoS One. 2023 May 30;18(5):e0285597. doi: 10.1371/journal.pone.0285597 (PMC10228790; doi:10.1371/journal.pone.0285597)
Supplement: Attachment — Submitted filename: Reviewer 2.pdf [file pone.0285597.s007.pdf]

Cancel | Save and Close

**PONE-D-23-00791**
**"Reassessing the adrenomedullin scavenging function of ACKR3 in lymphatic endothelial cells"**
**Original Submission**

[redacted] **(Reviewer 2)**

| | |
|---|---|
| **Reviewer Recommendation Term:** | Major Revision |
| **Rate Review:** | 0 |
| **Custom Review Question(s):** | **Response** |
| **Comments to the Author**<br><br>1. Is the manuscript technically sound, and do the data support the conclusions?<br><br>The manuscript must describe a technically sound piece of scientific research with data that supports the conclusions. Experiments must have been conducted rigorously, with appropriate controls, replication, and sample sizes. The conclusions must be drawn appropriately based on the data presented. | Yes |
| 2. Has the statistical analysis been performed appropriately and rigorously? | Yes |
| 3. Have the authors made all data underlying the findings in their manuscript fully available?<br><br>The PLOS Data policy requires authors to make all data underlying the findings described in their manuscript fully available without restriction, with rare exception (please refer to the Data Availability Statement in the manuscript PDF file). The data should be provided as part of the manuscript or its supporting information, or deposited to a public repository. For example, in addition to summary statistics, the data points behind means, medians and variance measures should be available. If there are restrictions on publicly sharing data—e.g. participant privacy or use of data from a third party—those must be specified. | Yes |
| 4. Is the manuscript presented in an intelligible fashion and written in standard English?<br><br>PLOS ONE does not copyedit accepted manuscripts, so the language in submitted articles must be clear, correct, and unambiguous. Any typographical or grammatical errors should be corrected at revision, so please note any specific errors here. | Yes |
| 5. Review Comments to the Author<br><br>Please use the space provided to explain your answers to the questions above. You may also include additional comments for the author, including concerns about dual publication, research ethics, or publication ethics. (Please upload your review as an attachment if it exceeds 20,000 characters) | In the study by Halin et Al., "Reassessing the adrenomedullin scavenging function of ACKR3 in lymphatic endothelial cells" the role of ACKR3 as scavenger of the vascular peptide hormone adrenomedullin (AM) was clarified. In this work, in vitro experiments were performed to evaluate the uptake of AM in primary human Lymphatic Endothelial Cells (LECs) and in ACKR3-overexpressing Human Epithelial Kidney (HEK) cells. The aim of this work was to verify a competition between the normal ACKR3-ligand CXCL11/12 and AM, to evaluate if this receptor can influence/reduce the AM proliferative effects on LECs. The Authors conclude that the AM does not compete with CXCL11/12 for ACKR3 binding, but it binds their canonical receptor (CALCRL and PAMP2/3. In my opinion this finding may have a relevance in this field, but major revisions are required.<br>The background should be improved, experimental procedures should be better described and rearranged. For example, first cell culture (including HEK cells), second the treatments and silencing on LECs, then the plasmid construction and transfection, finally the assay.<br>A few questions:<br>Why three type of LECs were used?<br>Why the coating was different between adLECs and nd- and jd-LECs?<br>The abbreviations should be uniformed and the same name should be used for the same protein, for example: ACKR3 was used throughout manuscript, but to indicate the antibody against this protein the CXCR/RDC-1 APC was used. The terms CXCL11/12-AF647 should be clarified as extended terms of CXCL; AF647 should be described as alexa flour 647; Atto 565 should be explained.<br>In the introduction, CCX771 should be described as an antagonist of ACKR3.<br>The description of the plasmids in the section "construction of expression plasmids for HEK293 cell transfection" should match with the description in the section "HEK293 cell transfection…"<br><br>In Figure1D, the error bars should be indicated in the graphic, which could be expanded to avoid the overlap between 37 °C ° and 37 °C + CCX771.<br>Please check and harmonize all the figures. For example, in fig 2D CXCL11/12 is reported as CXCl11/12; cell types are indicated in fig 3A, but not in fig 2. |
| 6. PLOS authors have the option to publish the peer review history of their article (what does this mean?). If published, this will include your full peer review and any attached files.<br><br>If you choose "no", your identity will remain anonymous but your review may still be made public.<br><br>**Do you want your identity to be public for this peer review?** For information about this choice, including consent withdrawal, please see our Privacy Policy. | No |
| **Confidential to Editor**<br><br>1. Do you have any potential or perceived competing interests that may influence your review? Please review our Competing Interests policy and declare any potential interests that you feel the Editor should be aware of when considering your review. If you have no competing interests, please write "I have no competing interests." | I have no competing interests |
| 2. Did you receive any assistance in preparing this review (e.g. from a post-doc or graduate student)? If yes, please include their name below. | no, I did not |
| 3. If accepted, do you think this submission should be highlighted on the PLOS ONE website? PLOS ONE does not evaluate manuscripts based on perceived significance or readership. We aim to provide tools for readers to filter and evaluate our publications. (optional) | |
| **Do you want to get recognition for this review on a Web of Science researcher profile?**<br><br>If you opt in, your Web of Science profile will automatically be updated to show a verified record of this review in full compliance with the journal's review policy. If you don't have a Web of Science profile, you will be prompted to create a free account. | Yes |

More Reviewer Details

Cancel | Save and Close
